# The C-reactive Protein-Albumin-Lymphocyte Index Predicts Survival Outcomes in Esophageal Cancer: A Meta-Analysis

**DOI:** 10.7759/cureus.93594

**Published:** 2025-09-30

**Authors:** Waqas Ul Bassar, Jahanzeb Akhtar, Nada Hasan Mukhtar Ismail, Zainab Shah, Manpreet Kaur Dhanjal, Sonalben Chaudhary, Naglaa G Ghobriel, Danish Allahwala

**Affiliations:** 1 Internal Medicine, Ayub Medical Teaching Institution, Abbottabad, PAK; 2 Pathology, Central Park Teaching Hospital, Lahore, PAK; 3 Internal Medicine, Misurata University, Misurata, LBY; 4 Hematopathology, Shifa International Hospital, Islamabad, PAK; 5 Medicine, Adesh Institute of Medical Sciences and Research, Ludhiana, IND; 6 Internal Medicine, Zydus Sitapur Hospital, Sitapur, IND; 7 Internal Medicine, University of Alexandria, Alexandria, EGY; 8 Nephrology, Fatima Memorial Hospital, Karachi, PAK

**Keywords:** c-reactive protein-albumin-lymphocyte index, esophageal cancer, meta-analysis, prognosis, survival

## Abstract

The C-reactive protein-albumin-lymphocyte (CALLY) index represents a novel composite biomarker integrating systemic inflammation, nutritional status, and immune function. This systematic review and meta-analysis aimed to evaluate the prognostic significance of the CALLY index in esophageal cancer patients. A comprehensive literature search was conducted across multiple databases from inception to August 2025, identifying studies that investigated the association between the CALLY index and survival outcomes in histologically confirmed esophageal cancer patients. The majority of studies employed retrospective cohort designs, with three being single-center studies and two utilizing multi-center approaches. The studies were conducted between 2024 and 2025, with four originating from Asian populations and one from a Western population. Meta-analysis revealed that patients with higher CALLY index values experienced significantly better overall survival compared to those with lower values (risk ratio (RR): 2.24, 95% CI: 1.71-2.94, p<0.001), despite moderate heterogeneity (I²=62%). Similarly, a higher CALLY index was associated with improved disease-free survival (RR: 1.99, 95% CI: 1.65-2.40, p<0.001) with no heterogeneity observed (I²=0%). Sensitivity analyses confirmed the robustness of these findings. The CALLY index demonstrates significant prognostic value in esophageal cancer, with lower values indicating worse outcomes. This composite biomarker may serve as a valuable tool for risk stratification and treatment planning, potentially guiding personalized therapeutic approaches. However, standardization of cut-off values and validation through prospective studies are needed to establish its clinical utility.

## Introduction and background

Esophageal cancer is recognized as one of the most aggressive malignancies worldwide, ranking seventh in incidence and sixth in cancer-related mortality [1]. In 2020 alone, there were an estimated 604,100 newly diagnosed cases and 544,076 deaths, underscoring the major clinical burden posed by this disease, given its poor prognosis and limited treatment strategies [2]. The overall five-year survival remains dismally low, around 15%-25%, primarily due to late presentation and the inherently aggressive course of the cancer [3]. The two principal histological types, esophageal squamous cell carcinoma and esophageal adenocarcinoma, differ in their epidemiology and molecular profiles, yet both are consistently linked with poor clinical outcomes [4].

Accurate prognostic assessment is crucial for optimal treatment planning, patient counseling, and clinical decision-making in esophageal cancer management. Traditional prognostic factors, including tumor stage, histological grade, and nodal involvement, while valuable, may not fully capture the complexity of cancer progression and patient outcomes [5]. Consequently, there has been growing interest in identifying novel biomarkers that can provide more precise prognostic information and potentially guide personalized treatment strategies.

The systemic inflammatory response and nutritional status have emerged as critical determinants of cancer prognosis. Chronic inflammation promotes tumor progression through various mechanisms, including angiogenesis, metastasis, and immune suppression [6]. Simultaneously, malnutrition and hypoalbuminemia are frequently observed in cancer patients and are associated with increased morbidity and mortality [7]. Lymphocytes, as key components of the adaptive immune system, play essential roles in anti-tumor immunity, and lymphopenia has been linked to poor outcomes in various malignancies [8].

The C-reactive protein-albumin-lymphocyte (CALLY) index represents a novel composite biomarker that integrates three fundamental aspects of cancer pathophysiology: systemic inflammation (CRP), nutritional status (albumin), and immune function (lymphocyte count) [9]. This index is calculated using the formula CALLY = (CRP/Albumin)/Lymphocyte count, where elevated values indicate a more unfavorable prognostic profile [10]. The CALLY index has demonstrated prognostic significance in several cancer types, including gastric, colorectal, and hepatocellular carcinomas [11-12].

Recent studies have investigated the prognostic value of the CALLY index specifically in esophageal cancer patients, yielding promising but heterogeneous results. Recent studies have investigated the prognostic value of the CALLY index specifically in esophageal cancer patients, yielding promising but heterogeneous results. However, these studies have shown inconsistencies across several key aspects, including variations in optimal cut-off thresholds for risk stratification, differences in patient demographics, and disease staging [13-14]. However, the clinical utility and reliability of this biomarker in esophageal cancer prognosis remain unclear due to varying study methodologies, patient populations, and outcome measures across individual studies.

Therefore, this systematic review and meta-analysis aims to comprehensively evaluate the association between the CALLY index and overall survival in patients with esophageal cancer. By synthesizing available evidence, we seek to determine whether the CALLY index can serve as a reliable prognostic tool for clinical practice and identify potential sources of heterogeneity among studies. Our findings may contribute to improved risk stratification and personalized treatment approaches for esophageal cancer patients.

## Review

Methods

This systematic review and meta-analysis was conducted according to the Preferred Reporting Items for Systematic Reviews and Meta-Analyses (PRISMA) 2020 guidelines [15].

Literature Search

A comprehensive literature search was performed across multiple electronic databases, including PubMed/Medical Literature Analysis and Retrieval System Online (MEDLINE), Excerpta Medica database (Embase), Web of Science, Cochrane Library, and Scopus from inception to 15 August 2025. The search strategy combined Medical Subject Headings (MeSH) and free-text terms related to esophageal cancer, CALLY index, and survival outcomes. The following search terms were used: ("esophageal cancer" OR "esophageal carcinoma" OR "esophageal neoplasm" OR "oesophageal cancer") AND ("C-reactive protein-albumin-lymphocyte" OR "CALLY" OR "CRP-albumin-lymphocyte") AND ("survival" OR "prognosis" OR "mortality" OR "outcome"). No language or publication date restrictions were applied. Additionally, reference lists of included studies and relevant review articles were manually searched to identify additional eligible studies. Conference abstracts and grey literature were also searched through Google Scholar and relevant conference proceedings. The search was performed by two authors. Any disagreement between two authors was resolved through discussion.

Study Selection

Studies were included if they involved patients with histologically confirmed esophageal cancer (both squamous cell carcinoma and adenocarcinoma), reported the CALLY index calculated as (CRP/Albumin)/Lymphocyte count, investigated the association between CALLY index and overall survival, provided sufficient data to calculate risk ratio (RR) with 95% confidence intervals (CI) or data that allowed for their calculation, were cohort studies (prospective or retrospective), or were case-control studies. Studies were excluded if they were case reports, case series, editorials, letters, or review articles; did not report overall survival as an outcome; did not provide sufficient data for statistical analysis; involved mixed cancer populations where esophageal cancer-specific data could not be extracted; were duplicate publications or studies with overlapping patient populations; or were animal or in vitro studies.

Two reviewers independently screened the titles and abstracts of all identified articles based on predefined inclusion and exclusion criteria. Full texts of studies deemed potentially eligible were subsequently assessed by the same reviewers. Any conflicts in selection were resolved through consultation with a third reviewer. The overall study selection process was illustrated using a PRISMA flow diagram.

Data Extraction

Data extraction was performed independently by two reviewers using a standardized data extraction form. The following information was extracted from each included study: study characteristics including first author, publication year, country, and study design; patient characteristics including sample size, age, sex distribution, histological type, tumor stage, and treatment modalities; CALLY index details including cut-off values, calculation method, and timing of measurement; outcome measures including overall survival data and disease-free survival, and median follow-up time. Any discrepancies in data extraction were resolved through discussion between reviewers.

Quality Assessment

The quality of the included studies was evaluated using the Newcastle-Ottawa Scale (NOS) for cohort studies [16]. This tool assesses three domains: selection of study participants (up to 4 points), comparability between groups (up to 2 points), and outcome assessment (up to 3 points), with a total possible score of 9. Studies achieving a score of 7 or higher were classified as high quality, those scoring 5-6 as moderate quality, and those scoring below 5 as low quality. Two reviewers independently performed the quality assessment, and any discrepancies were resolved through discussion and consensus.

Data Analysis

All statistical analyses were performed using Review Manager (RevMan) version 5.4 software provided by the Cochrane Collaboration, London, United Kingdom. RRs with 95% CI were calculated as the primary effect measure for dichotomous survival outcomes comparing high CALLY versus low CALLY index groups, with the CALLY index dichotomized according to cut-off values established by individual studies. A random-effects model was employed as the primary analytical approach to account for expected clinical and methodological heterogeneity across studies, particularly given the variation in CALLY cut-off values ranging from 1.7 to 2.55 across included studies. Statistical heterogeneity was evaluated using the I² statistic and Cochran's Q test, with heterogeneity classified as low (I² ≤ 25%), moderate (I² = 26-50%), substantial (I² = 51-75%), or considerable (I² > 75%), and P-values < 0.10 for the Q test considered indicative of significant heterogeneity. Forest plots were generated using RevMan to visually display individual study results and pooled estimates. The p-value cut-off was kept at 0.05.

Results

**Figure 1:**
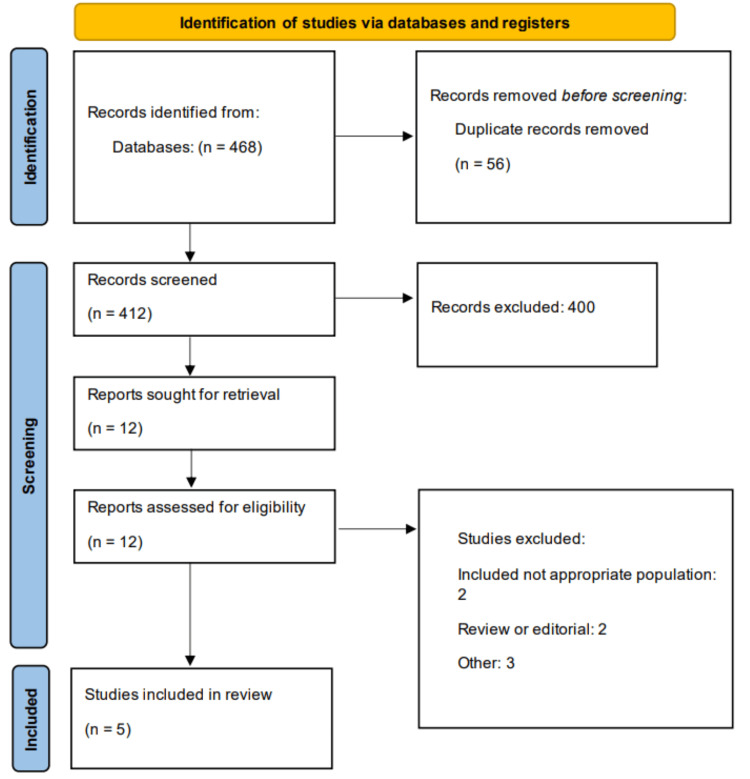
A PRISMA flowchart outlining the study selection process PRISMA: Preferred Reporting Items for Systematic Reviews and Meta-Analyses

The systematic literature search across multiple databases yielded a total of 468 records. Following title and abstract screening, 12 full-text articles were assessed for eligibility according to the predefined inclusion and exclusion criteria. Ultimately, five studies met the inclusion criteria and were included in the meta-analysis, comprising a total of 2,192 esophageal cancer patients. The included studies were conducted between 2024 and 2025, with sample sizes ranging from 104 to 657 patients. Five studies originated from Asian populations (three from China and two from Japan). The majority of studies employed retrospective cohort designs, with three being single-center studies and two utilizing multi-center approaches. The study selection process is detailed in the PRISMA flow diagram (Figure 1), and the characteristics of included studies are summarized in Table 1. Table 2 presents the quality assessment of included studies.

**Table 1:** Characteristics of the included studies CALLY: C-reactive protein-albumin-lymphocyte; NR: not reported

Study ID	Region	Study Design	Study Period	Sample Size	Number with Higher CALLY	Cut-Off Used	Mean Age (Years)	Male (n)	Blood Sampling Timing	TNM Stage (III/IV)
Aoyama et al., 2024 [17]	Japan	Retrospective cohort study	2005-2020	180	88	>=5	60	112	NR	112
Feng et al., 2024 [13]	China	Retrospective cohort study	2013-2015	318	171	>1.7	59.4	213	One week prior to surgery	115
Jia et al., 2025 [18]	China	Prospective cohort study	2013-2023	518	147	>= 2.51	69	462	Within 24 hours of admission after overnight fasting	345
Ma et al., 2024 [19]	Japan	Retrospective cohort study	2008-2018	146	102	>2.40	69	123	Within one week before surgical resection	42
Meng et al., 2025 [20]	China	Retrospective cohort study	2016-2024	553	296	>2.55	64.8	456	At 6 a.m. on the day after hospital admission	313

**Table 2:** Quality assessment of the included studies using Newcastle Ottawa Scale The maximum score for selection is 4. The maximum score for comparability is 2. The maximum score for the outcome is 3.

Study ID	Selection	Comparability	Outcome	Quality Rating
Aoyama et al., 2024 [17]	4	2	3	High
Feng et al., 2024 [13]	4	2	3	High
Jia et al., 2025 [18]	4	2	3	High
Ma et al., 2024 [19]	3	1	3	High
Meng et al., 2025 [20]	4	2	3	High

Meta-analysis of Outcomes

**Figure 2:**
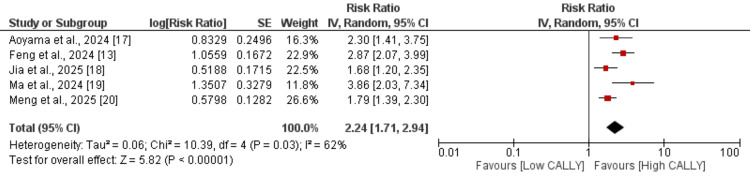
Effect of CALLY on overall survival CALLY: C-reactive protein-albumin-lymphocyte References [13, 17-20]

Overall survival: Five studies were included in the pooled analysis to compare the overall survival between high CALLY and low CALLY patients with esophageal cancer. As shown in Figure 2, subjects with high CALLY were associated with increased overall survival compared to the subjects with lower CALLY (RR: 2.24, 95% CI: 1.71, 2.94), and this difference was statistically significant. High heterogeneity was reported among the study results (I^2^: 62%). Table 3 presents the sensitivity analysis. Our analysis showed that patients with a high CALLY index consistently experienced good survival outcomes compared with those with lower values. Although some heterogeneity was observed across studies, the direction of effect remained uniform, supporting the role of lower CALLY as a predictor of poor prognosis. Sensitivity analyses further confirmed the stability of the findings, suggesting that the association between low CALLY and adverse survival is robust and not dependent on any single study.

**Table 3:** Sensitivity analysis (overall survival) RR: risk ratio

Study ID	RR (95% CI)	I^2^
Aoyama et al., 2024 [17]	2.25 (1.62, 3.13)	71%
Feng et al., 2024 [13]	2.05 (1.55, 2.71)	50%
Jia et al., 2025 [18]	2.45 (1.77, 3.39)	63%
Ma et al., 2024 [19]	2.08 (1.61, 2.68)	56%
Meng et al., 2025 [20]	2.44 (1.74, 3.43)	60%

**Figure 3:**
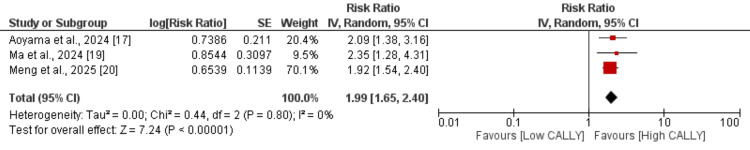
Effect of CALLY on disease-free survival CALLY: C-reactive protein-albumin-lymphocyte References [17, 19-20]

Disease-free survival: Three studies were included in the pooled analysis to compare the disease progression survival between high and low CALLY subjects with esophageal cancer, and the results are demonstrated in Figure 3. The pooled analysis showed that subjects with high CALLY reported better disease-free survival compared to their counterparts (RR: 1.99, 95% CI: 1.65, 2.40). No heterogeneity was reported among the study results (I²: 0%).

Discussion

This study compiles the available evidence on the prognostic significance of the CALLY index in patients with esophageal cancer. Following a comprehensive literature search, five studies evaluating the role of CALLY in esophageal cancer were included. The findings of this meta-analysis demonstrated that patients with a higher CALLY index had a significantly reduced risk of unfavorable disease-free survival and overall survival outcomes. Sensitivity analysis further reinforced the robustness of the results, as the exclusion of individual studies did not alter the significance of overall survival findings. Similarly, Li et al. [11], in a pooled analysis of six studies on gastric cancer, reported that a lower CALLY index served as an independent prognostic factor for both overall survival and progression-free survival. Beyond gastrointestinal cancers, CALLY has also shown prognostic value in other malignancies. For instance, in patients with metastatic or recurrent head and neck cancer treated with pembrolizumab, a low CALLY index was linked to significantly shorter overall survival [21]. Likewise, Mutlucan et al. [22] observed lower CALLY scores among deceased glioblastoma patients compared to survivors, highlighting its role as a prognostic marker. Furthermore, the preoperative CALLY index has been identified as an important predictor, independently associated with poorer outcomes in gastric cancer patients undergoing gastrectomy [23]. Despite the distinct biological characteristics of gastric and esophageal cancers, the CALLY index may demonstrate similar prognostic value in both malignancies due to shared underlying mechanisms. Both cancer types commonly induce systemic inflammatory responses characterized by elevated inflammatory markers and cytokine dysregulation, which are reflected in the inflammatory components of the CALLY index. Additionally, both gastric and esophageal cancers frequently lead to malnutrition and muscle wasting through similar pathophysiological pathways, including mechanical obstruction, decreased oral intake, and cancer-associated cachexia [22].

A low CALLY index embodies a pathophysiological triad of systemic inflammation, malnutrition, and immune suppression, all of which contribute to tumor progression and unfavorable outcomes in patients with esophageal cancer [24]. Elevated CRP reflects persistent inflammation, which fosters a tumor-supportive microenvironment through the release of pro-inflammatory cytokines such as IL-6 and TNF-α, thereby enhancing angiogenesis, metastasis, and resistance to therapy [25]. Reduced albumin levels signify protein-energy malnutrition and impaired hepatic protein synthesis, leading to compromised wound healing, weakened immunity, and diminished tolerance to treatment, issues that are especially pronounced in esophageal cancer due to dysphagia-related nutritional decline [26]. As a result, individuals with a low CALLY index experience markedly higher incidences of postoperative complications, treatment-related adverse effects, and faster disease progression [20]. By integrating these factors, the CALLY index provides a more holistic reflection of the host-tumor interaction than single biomarkers, which accounts for its superior prognostic utility and highlights potential targets for combined strategies such as anti-inflammatory interventions, nutritional optimization, and immune-based therapies [27].

It can be argued that cancer prognosis is strongly influenced by factors such as disease stage, presence of comorbidities, metastatic status, and treatment modalities. These variables likely contribute substantially to the heterogeneity observed across studies. Notably, the reported cut-off values for the CALLY index varied considerably, ranging from 1.7 to 2.55. Despite this variation, each study identified an optimal threshold within its own cohort and still arrived at consistent findings. Such results are both noteworthy and in line with previous meta-analyses [28, 29] on other inflammatory biomarkers, where the most appropriate prognostic cut-off has yet to be clearly defined. These findings should be interpreted with caution until an evidence-based universal threshold is validated. We suggest that additional research is needed to establish the optimal CALLY threshold for prognostic assessment.

The clinical utility of the CALLY index extends beyond prognostication to informing treatment decisions and resource allocation. Patients with a low CALLY index represent a high-risk population that may benefit from intensified interventions, including preoperative nutritional optimization, anti-inflammatory therapies, and enhanced surveillance protocols. Conversely, patients with a high CALLY index may be candidates for treatment de-escalation or alternative therapeutic approaches.

This meta-analysis is subject to several limitations. To begin with, only five studies were eligible for inclusion. Additionally, subgroup analyses based on factors such as cancer stage, histological type, treatment modalities, and treatment response could not be performed, mainly due to the limited number of available studies. Additionally, we were not able to perform meta-regression as the number of included studies was low. The predominance of retrospective designs among the included studies raises concerns of selection bias and restricts the ability to infer causality. Therefore, prospective studies are required to validate the prognostic value of the CALLY index and to assess its influence on treatment strategies and patient outcomes. Finally, since most of the included studies originated from China, the findings may not be broadly generalizable at this stage. Several regional differences could potentially influence the applicability of the CALLY index to non-Chinese populations, including variations in esophageal cancer epidemiology (with squamous cell carcinoma predominant in China versus adenocarcinoma in Western countries), differences in treatment protocols and surgical approaches, and distinct baseline nutritional status and inflammatory profiles across ethnic groups. Therefore, validation studies in diverse populations with different epidemiological backgrounds, treatment approaches, and nutritional contexts are essential before the CALLY index can be considered a universally applicable prognostic tool in esophageal cancer management.

Future research should focus on several key areas: (1) standardization of CALLY cut-off values across different populations and treatment settings; (2) investigation of dynamic CALLY changes during treatment and their prognostic implications; (3) evaluation of CALLY-guided interventions, including nutritional support and anti-inflammatory therapies; (4) integration of the CALLY index with molecular biomarkers and advanced imaging techniques for enhanced prognostication; and (5) assessment of the CALLY index in the context of emerging immunotherapies and targeted treatments

## Conclusions

This systematic review and meta-analysis provides compelling evidence that the CALLY index serves as a significant prognostic biomarker in esophageal cancer patients. Our findings demonstrate that patients with higher CALLY index values experience substantially better overall survival and disease-free survival outcomes compared to those with lower values. The CALLY index effectively integrates three critical pathophysiological components, systemic inflammation, nutritional status, and immune function, offering a comprehensive assessment tool superior to individual biomarkers. Despite moderate heterogeneity in overall survival analysis, sensitivity analyses confirmed the robustness of these findings across all included studies. The clinical utility of the CALLY index extends beyond prognostication to informing treatment decisions and identifying high-risk patients who may benefit from intensified interventions. However, standardization of optimal cut-off values and validation through large-scale prospective studies are essential to establish its routine clinical implementation in esophageal cancer management.
